# Supplementary material for: Integrated genomic and proteomic analysis of the mouse-adapted Staphylococcus aureus strain JSNZ
Source: Curr Res Microb Sci. 2025 Oct 13;9:100489. doi: 10.1016/j.crmicr.2025.100489 (PMC12554148; doi:10.1016/j.crmicr.2025.100489)
Supplement: Supplementary file 1 [file mmc1.pdf]

<u>Supplementary data</u>

Integrated genomic and proteomic analysis of the mouse-adapted *Staphylococcus aureus strain* JSNZ

Hannes Wolfgramm, Larissa Milena Busch, Jöran Tebben, Henry Mehlan, Lisa Hagenau, Thomas Sura, Tilly Hoffmüller, Elisa Bludau, Manuela Gesell Salazar, Alexander Reder, Stephan Michalik, Leif Steil, Kristin Surmann, Ulrike Mäder, Silva Holtfreter, Uwe Völker*

<u>This PDF includes:</u>

Figure S1. Genomic comparison of genomic islands (GI), island-like structures and repetitive gene clusters in the JSNZ genome to the four other CC88 genomes considered in this study.

Figure S2. Protein sequence alignment of cell-wall-anchored protein SasG orthologs.

Figure S3. Assignment of the two JSNZ encoded HsdS (JSNZ_000367 and JSNZ_001801) to the respective target recognition domains (TRDs).

Figure S4. Schematic visualization of the novel Type IV restriction system, classified as a coiled-coil tandem (CoCoNuT) of subtype I-B.

Figure S5. Abundances of proteins identified in JSNZ exponential and stationary cultures grown in TSB or RPMI.

Figure S6. Proteomic coverage of genomic islands and island-like structure depicted by protein abundance assigned to corresponding genes.

Figure S7. Abundance of detected proteins encoded by annotated pseudogenes in the JSNZ genome.

Figure S8. Enrichment of protein sets comparing growth phases.

Figure S9. Enrichment of protein sets comparing media.

Figure S10. Protein sequence alignment of Jep to the Spls encoded in JSNZ and USA300_FPR3757.

Table S1. Summary of sequencing read statistics.

Table S2. Reference integrase sequences used for phage classification.

TableS3. Information on reversed phase liquid chromatography (RPLC).

TableS4. Information on data independent analysis (DIA) mass spectrometry.

<u>Additional files:</u>

Supplementary data 1. Information on annotated genes, corresponding orthologs, detected abundance of corresponding proteins and statistics.

> **Supplementary table 1-1.** Curated annotation of the JSNZ genome including meta information.
>
> **Supplementary table 1-2.** Regulon prediction of annotated JSNZ genes.
>
> **Supplementary table 1-3.** Protein sequence similarity of the annotated JSNZ genes to orthologous genes of *S. aureus* strains muCC88d, AUS0325, 21343, COL, NCTC8325, Newman, and USA300.
>
> **Supplementary table 1-4.** Mass spectrometric detected and quantified proteins in JSNZ culture samples grown in TSB and RPMI.
>
> **Supplementary table 1-5.** Consistently high-abundant proteins of JSNZ.
>
> **Supplementary table 1-6.** ROPECA statistics on protein alterations between exponential and stationary growth phase per medium.
>
> **Supplementary table 1-7.** ROPECA statistics on relative protein alterations between JSNZ grown in TSB and RPMI per growth phase.

Supplementary data 2.

> **Supplementary table 2.** Overview on JSNZ genes classified as virulence or resistance related.

Supplementary data 3. Information on occurance of mouse-specific mutations in JSNZ, peptide coverage of detected proteins assigned to pseudogenes and proteins with unexpected localization.

> **Supplementary table 3-1.** Occurrence of mouse-associated, non-phage located mutations in the JSNZ genome.
>
> **Supplementary table 3-2.** Proteins with unexpected distribution between cellular fraction and supernatant contradicting predicted localization (DeepLocPro).
>
> **Supplementary table 3-3.** Peptide coverage of the five pseudogene derived proteins detected by mass spectrumetry.

Supplementary data 4. Graphical summary of the JSNZ genome, orthologous genes in the AureoWiki strains, and proteomic coverage across all conditions.

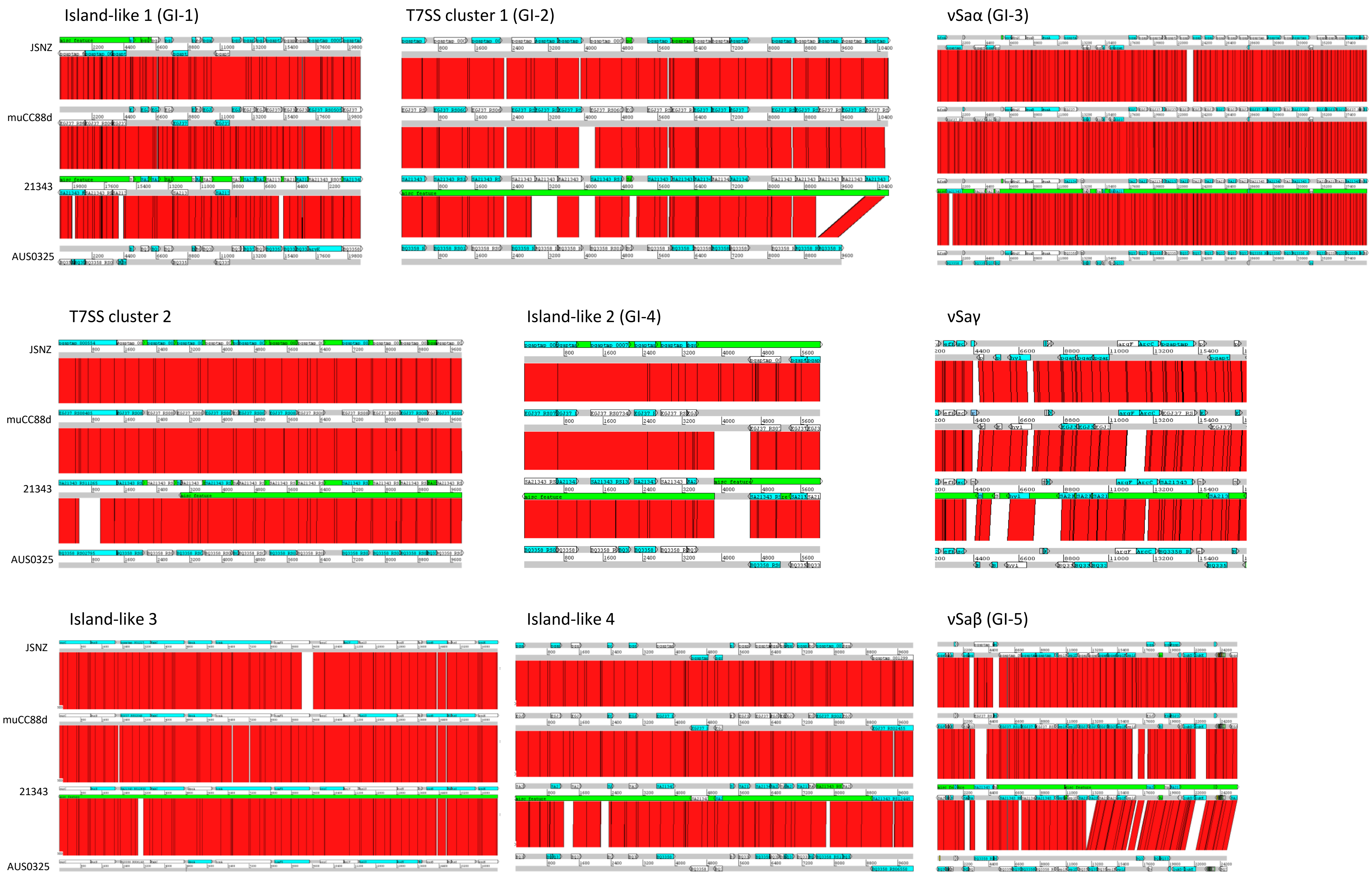

Figure S1. Genomic comparison of genomic islands (GI), island-like structures and repetitive gene clusters in the JSNZ genome to the three other CC88 genomes considered in this study. Genomic structure was compared with the Artemis Comparison Tool, identity level was set to 95%. GIs defined as specific for CC88 according to Kpeli *et al.* (2017) are mentioned in brackets. The comparison of ΦLabRodCC88_3 is depicted in Fig. 1C of the main text.

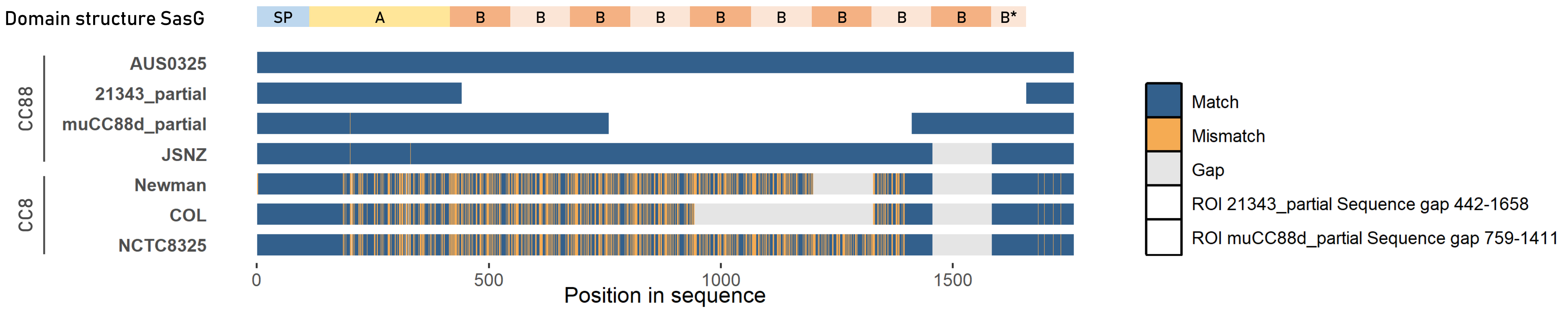

Figure S2. Protein sequence alignment of cell wall-anchored protein SasG orthologs. Amino acid mismatches (yellow), gaps (grey) and premature stop codons (dark red) are depicted. Domain structure is indicated above the alignment. Due to the repetitive structure of B domains, short-read genome sequences of isolates 21343 and muCC88d exhibit a sequencing gap interrupting the *sasG* sequence. SP signal peptide, B* truncated B domain.

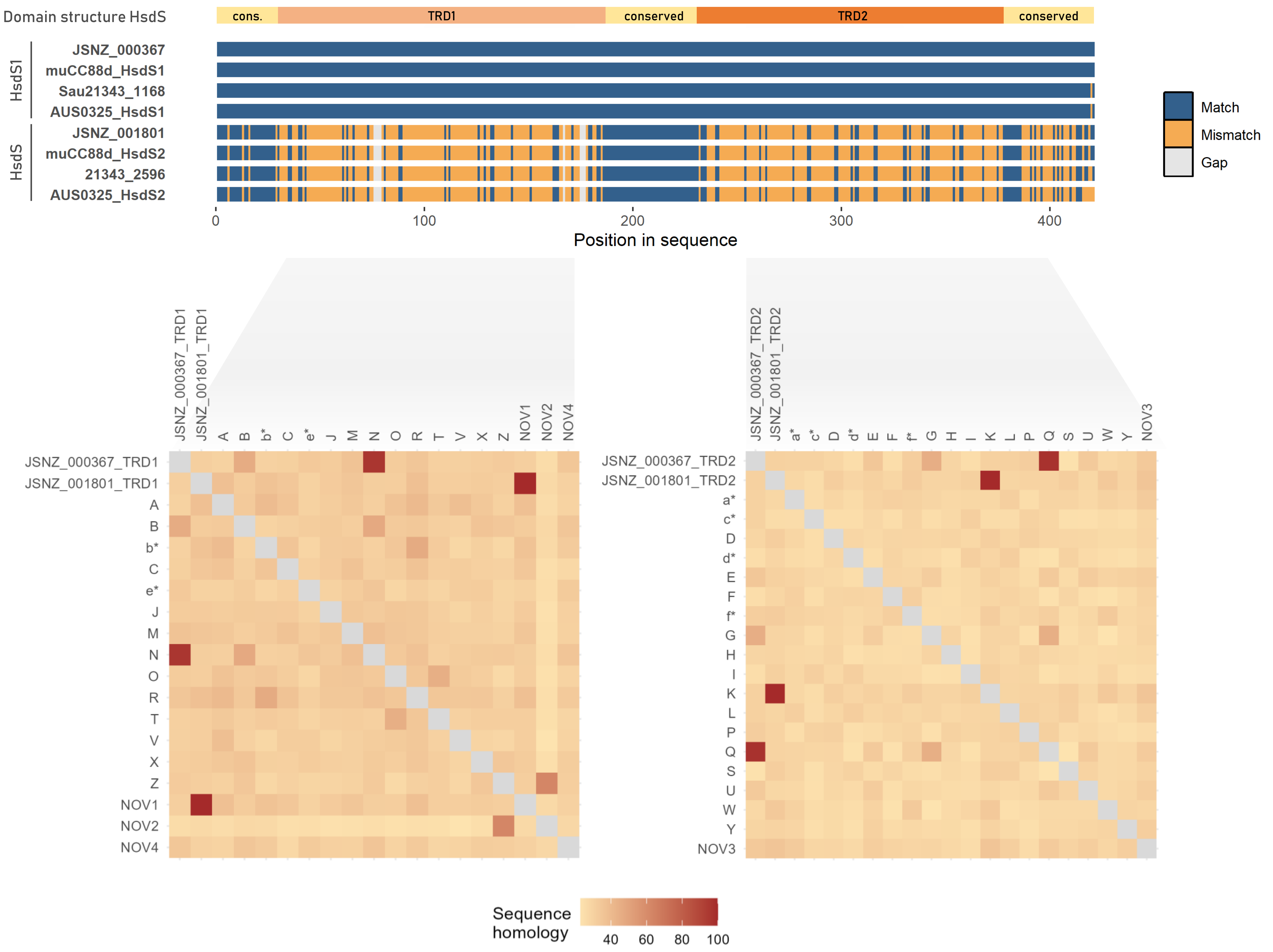

Locus tag	Annotation	TRDs	Recognition sequence
JSNZ_000367	HsdS1	N-Q	ACC-5-RTGT
JSNZ_001801	HsdS	NOVEL1-K	GAG-6-TCG

Figure S3. Assignment of the two JSNZ encoded HsdS (JSNZ_000367 and JSNZ_001801) to the respective target recognition domains (TRDs). (Top) Protein sequence alignment of both HsdS copies and their orthologs in the other analysed CC88 isolates. Protein domain structure is depicted above. (Bottom) Sequence similarity matrix comparing TRD1 and TRD2 of JSNZ_000367 and JSNZ_001801 to the reference sequences published by Cooper *et al.* (2017). Each of the four TRDs can be clearly assigned to a reference TRD, as indicated by the distinct red squares. The resulting TRD combination with according recognition sequence is listed in the table. Underlined nucleotides are methylation targets.

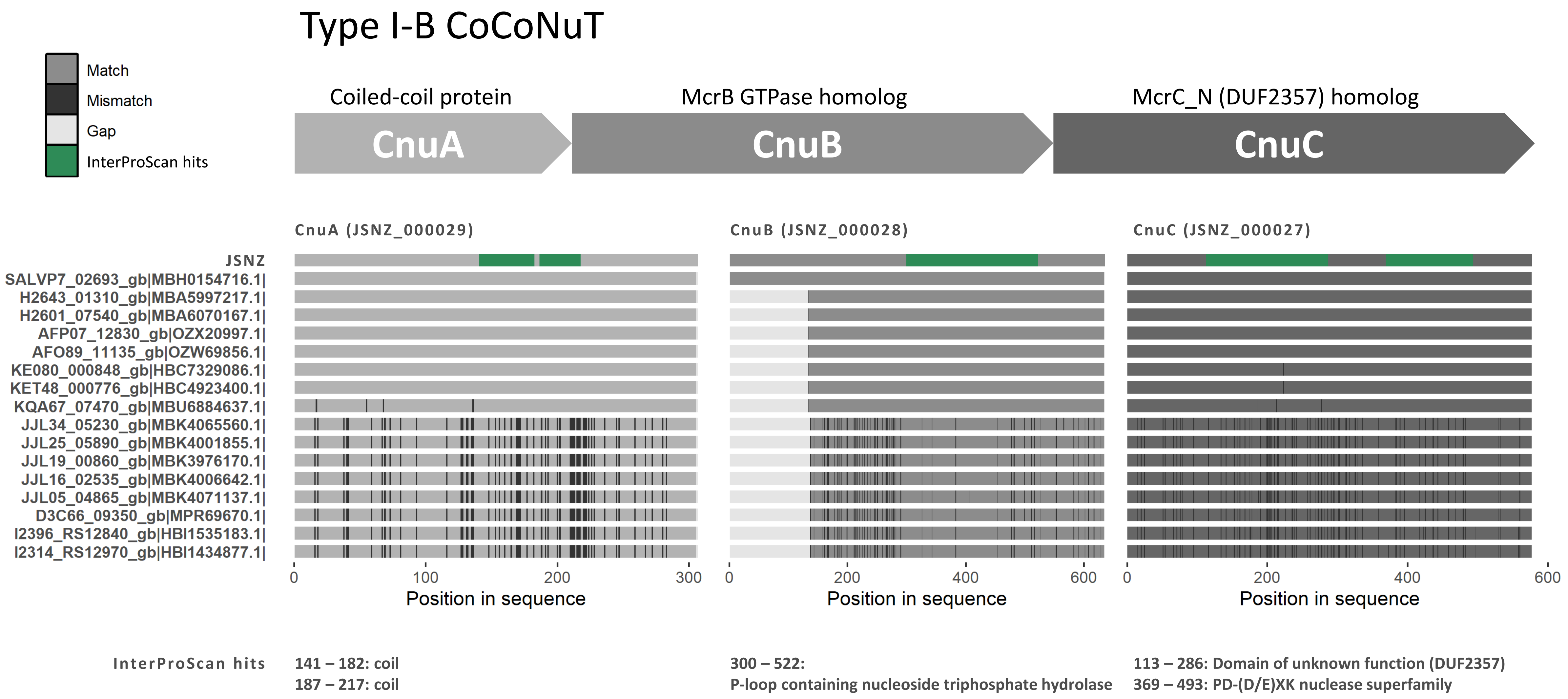

Figure S4. Schematic visualization of the novel type IV restriction system, classified as a coiled-coil tandem (CoCoNuT) of subtype I-B. Classification was validated by (i) protein sequence alignment of JSNZ_000027 to JSNZ_000029 with representative CnuA, CnuB and CnuC sequences (Bell *et al.*, 2024) and (ii) identification of typical domains *via* InterProScan analysis, highlighted green in the upper JSNZ sequence and detailed below the alignment.

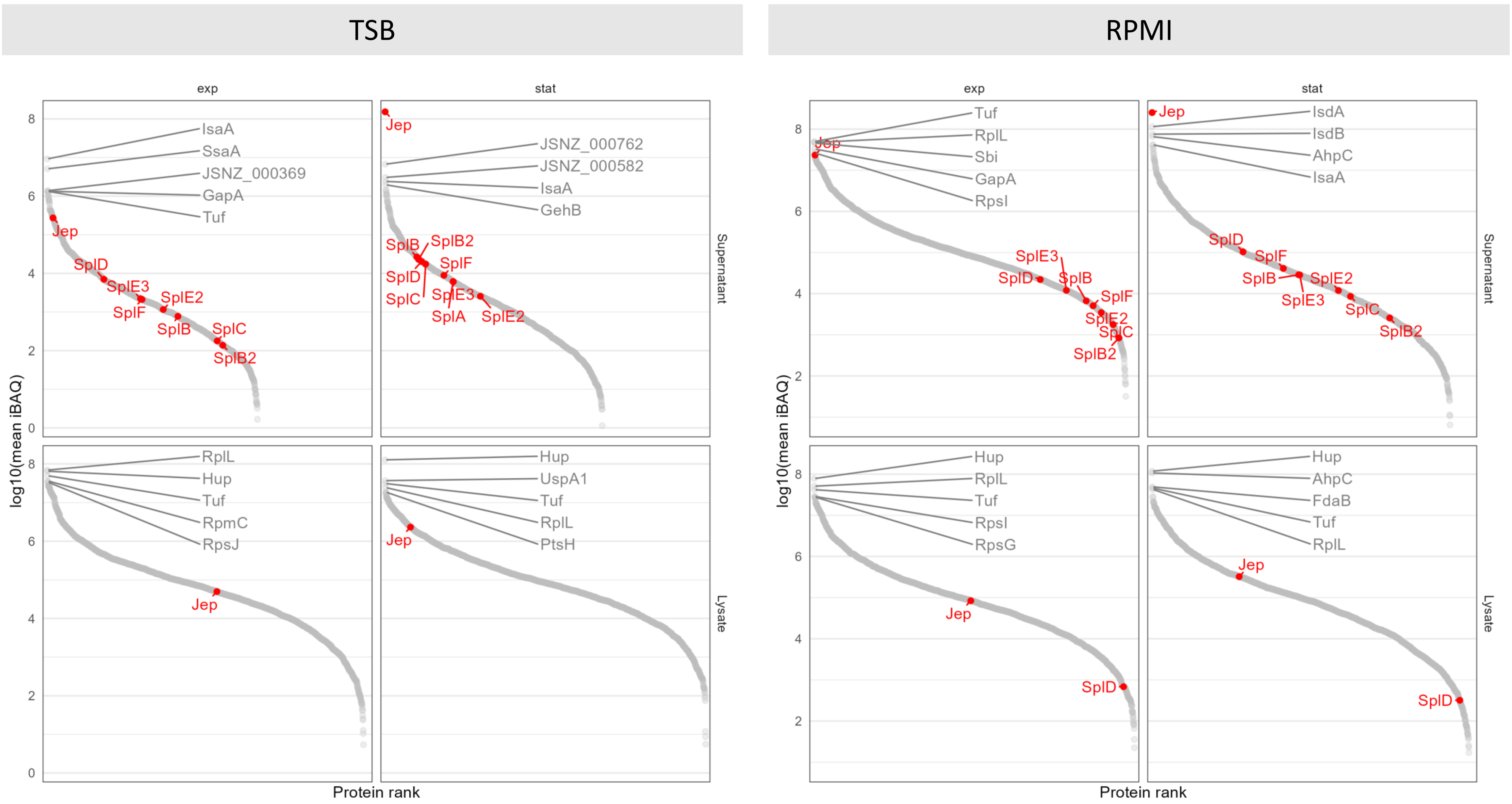

Figure S5. Abundances of proteins identified in JSNZ exponential and stationary cultures grown in TSB or RPMI. Proteins separately ranked by iBAQ. Secreted serine-protease like proteins (Spls) and JSNZ extracellular protease (Jep) are labelled in red, top five most abundant proteins per sample are labelled in grey.

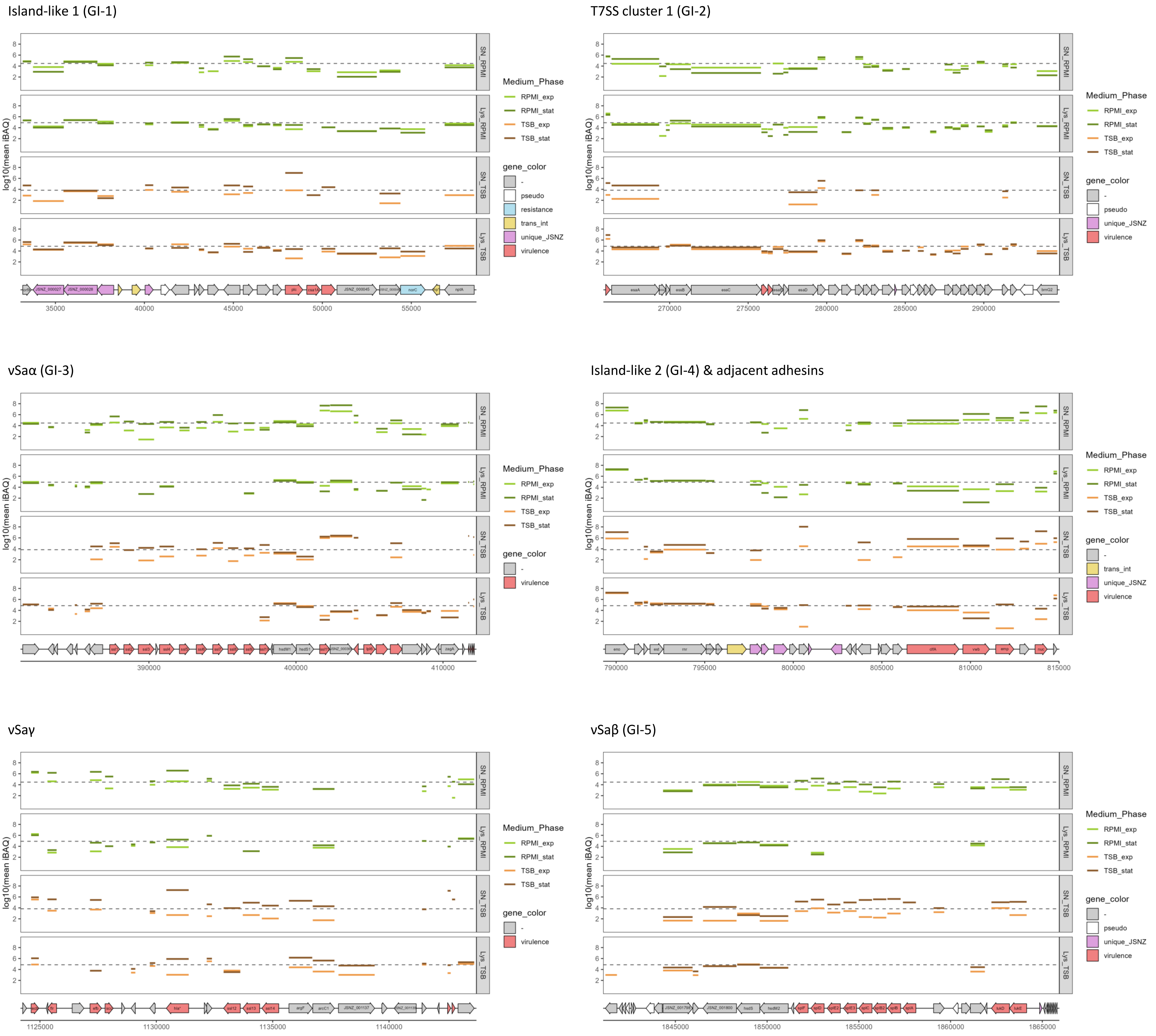

Figure S6. Proteomic coverage of genomic islands and island-like structure depicted by protein abundance assigned to corresponding genes. Islands were selected based on contained virulence genes. The annotated genome region is shown with genome position in base pairs. Median iBAQ per medium and fraction is indicated by a dashed line, protein iBAQ lines are coloured based on condition. iBAQ calculation based on four bioreplicates. ΦLabRodCC88_3 is depicted in Fig. 3F of the main text.

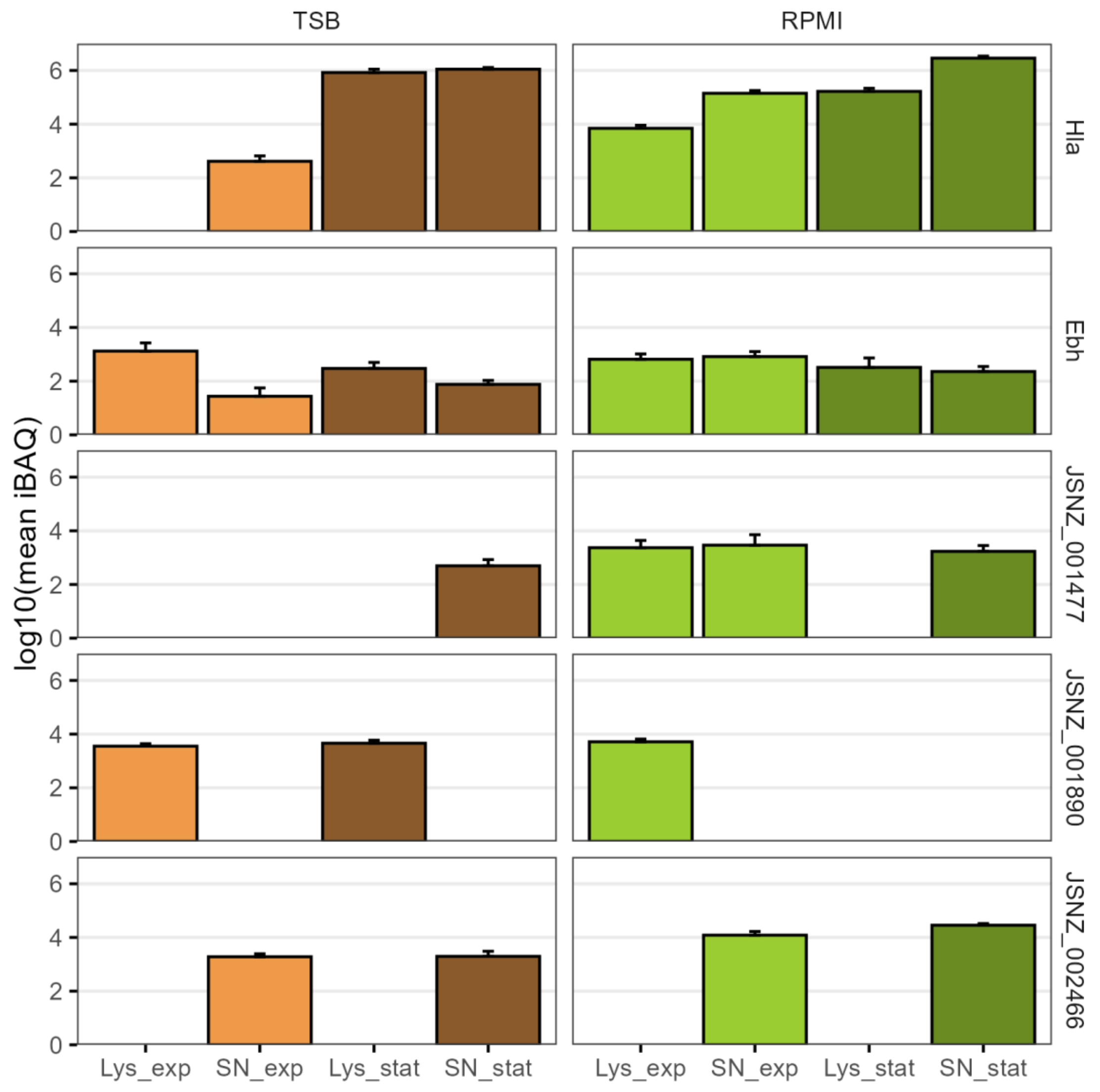

Figure S7. Abundance of detected proteins encoded by annotated pseudogenes in the JSNZ genome. iBAQs are calculated based on four bioreplicates, standard deviation is shown by error bars.

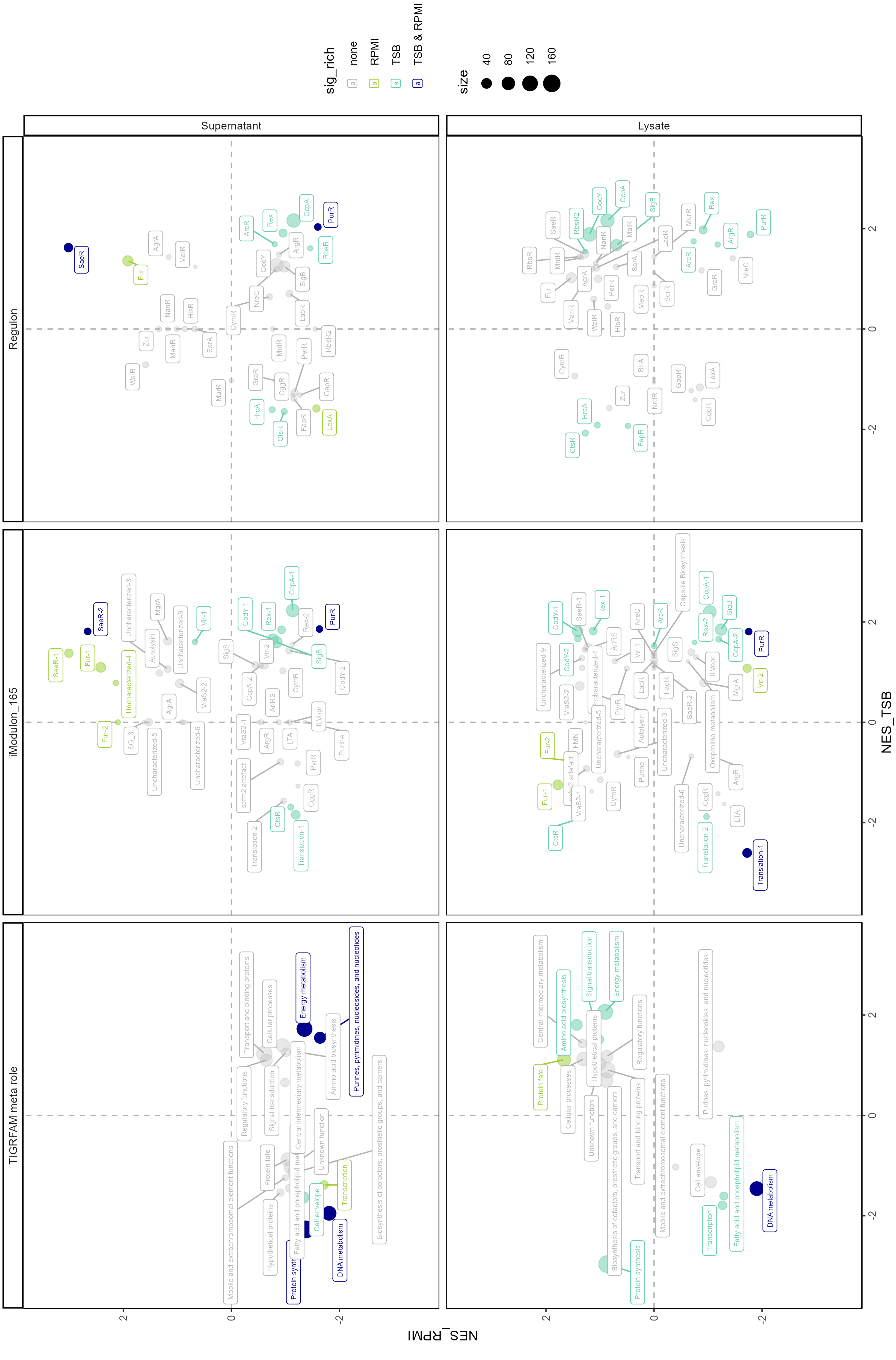
Supernatant
Lysate
Regulon
iModulon_165
TIGRFAM meta role
NES_TSB
NES_RPMI
sig_rich
none
RPMI
TSB
TSB & RPMI
size
40
80
120
160

Figure S8

Figure S8. Enrichment of protein sets comparing growth phases. GSEA statistics for proteome alteration from exponential to stationary growth phase per medium. Proteins were grouped to sets according to TIGRAFM meta role (left), assigned iModulon (center), and regulon (right), respectively. Points are coloured based on significant enrichment (adjusted p-value <0.05) in one or both respective comparisons, point size is relative to protein set size. Legends are centrally collected. Explanatory, schematic plots are included to aid data interpretation. This figure is the fully annotated version of Fig. 4A from the main text. NES = normalized enrichment score.

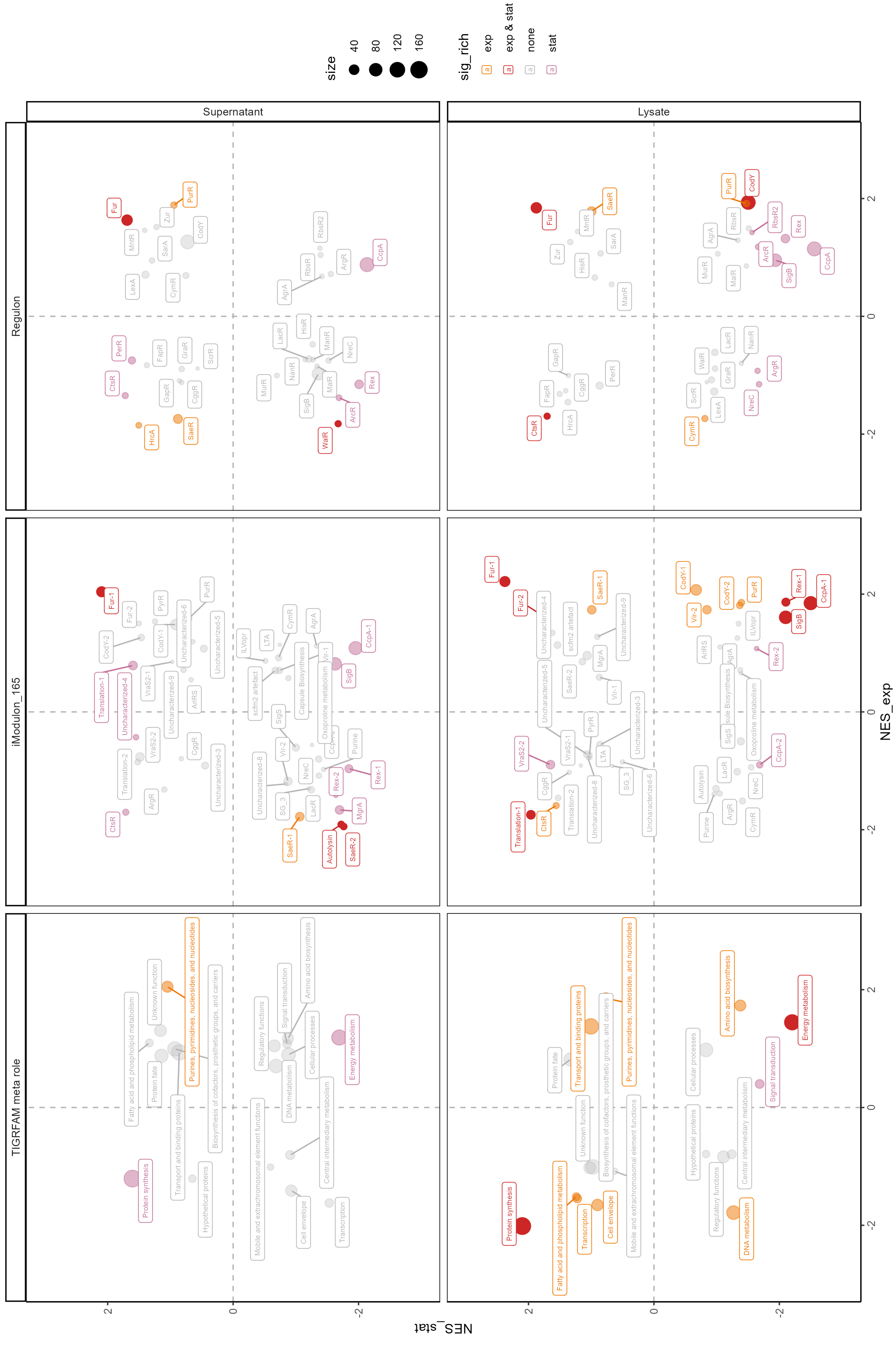
TIGRFAM meta role
iModulon_165
Regulon
Supernatant
Lysate
NES_exp
NES_stat
size
40
80
120
160
sig_rich
exp
exp & stat
none
stat

Figure S9

Figure S9. Enrichment of protein sets comparing media. GSEA statistics for relative proteome alteration from TSB to RPMI per growth phase. Proteins were grouped to sets according to TIGRAFM meta role (left), assigned iModulon (center), and regulon (right), respectively. Points are coloured based on significant enrichment (adjusted p-value <0.05) in one or both respective comparisons, point size is relative to protein set size. Legends are centrally collected. Explanatory, schematic plots are included to aid data interpretation. This figure is the fully annotated version of Fig. 4B from the main text. NES = normalized enrichment score.

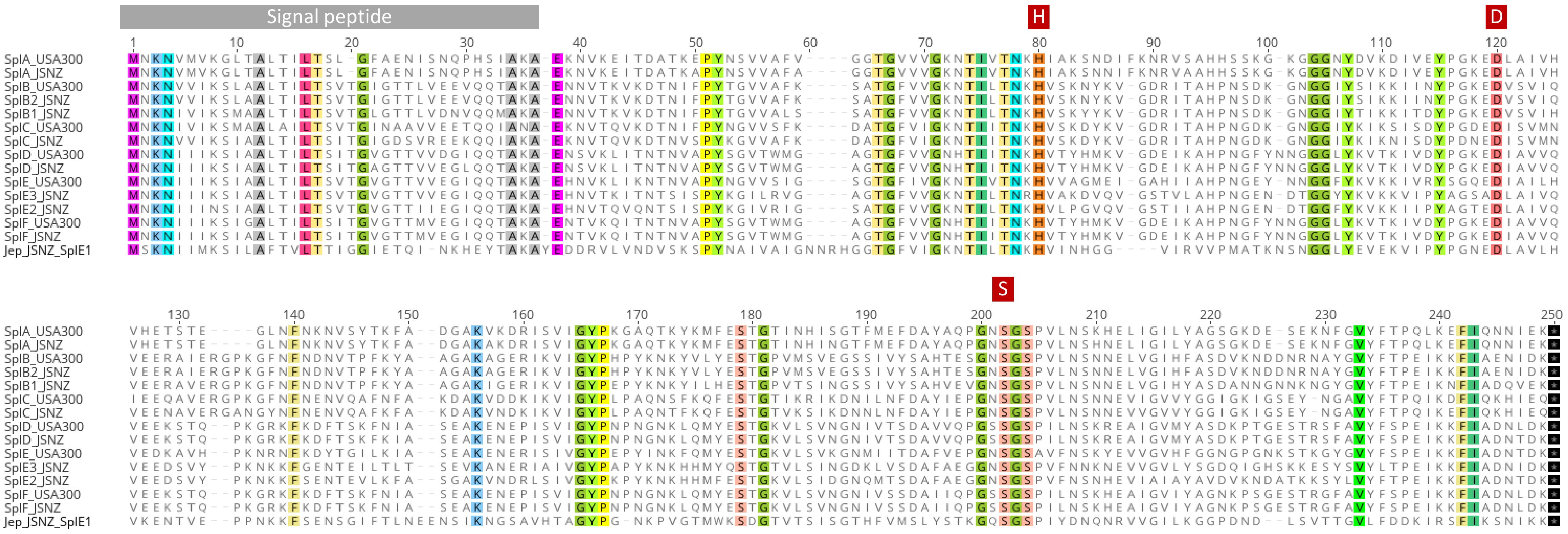

Figure S10. Protein sequence alignment of Jep (bottom sequence) to the Spls encoded in JSNZ and USA300_FPR3757. Conserved amino acids are coloured, signal peptide (grey) and catalytic triade (red) are indicated above the sequences. The alignment is directly visualized in Geneious Prime.

Supplementary Tables

Table S1. Summary of sequencing read statistics.

	Raw reads	Trimmed reads
ONT		
n	102868	62643
Total length	726.4 Mb	653.8 Mb
Mean length	7.1 kb	10.4 kb
Median length	4.0 kb	7.2 kb
N 50	12.6 kb	14.2 kb
Min length	43 bp	2,468 bp
Max length	139.0 kb	139.0 kb
coverage		240.4x
Illumina (paired reads)		
n	5641601	5579151
length	2x 150 bp	2x 36 bp to 150 bp
coverage		ca. 600x

Table S2. Reference integrase sequences used for phage classification according to Goerke *et al.* (2009).

Class	Exemplary phage	NCBI accession
Sa1int	*Staphylococcus* phage phiETA	NP_510895.1
Sa1int	*Staphylococcus* phage 55	YP_240491.1
Sa2int	*Staphylococcus* phage PVL	NP_058467.1
Sa3int	*Staphylococcus* phage phi 13	NP_803356.1
Sa4int	*Staphylococcus* phage phiSauS-IPLA35	YP_002332364.1
Sa5int	*Staphylococcus* phage 29	YP_240566.1
Sa6int	*Staphylococcus* phage 88	YP_240703.1
Sa7int	*Staphylococcus* phage 52A	AAX91804.1
Sa8int	*Staphylococcus* phage 53	YP_239679.1
Sa9int	*Staphylococcus* phage vB_SauS-phiIPLA88	YP_002332477.1
Sa10int	*Staphylococcus* phage 96	AAX91428.1
Sa11int	*Staphylococcus* phage 37	AAX91273.1
Sa12int	*Staphylococcus* phage EW	YP_240184.1

Table S3. Information on reversed phase liquid chromatography (RPLC).

Instrument	Ultimate 3000 RSLC (Thermo Scientific)
Trap column	75 µm inner diameter, packed with 3 µm C18 particles<br>(Acclaim PepMap100, Thermo Scientific)
Analytical column	Accucore 150-C18, (Thermo Fisher Scientific)<br>25 cm x 75 µm, 2,6 µm C18 particles, 150 Å pore size
Buffer system	binary buffer system consisting of 0.1% acetic acid in HPLC-grade water (solvent A) and 100% ACN in 0.1% acetic acid (solvent B)
Flow rate	300 nl/min
Gradient	0min-2% B<br>2min-5% B<br>10min-7% B<br>70min-25% B<br>75min-40% B<br>77min-90% B<br>83min-90% B<br>85min-2% B<br>95min-2% B
Column oven temperature	40°C

Table S4. Information on data independent analysis (DIA) mass spectrometry.

Instrument	Orbitrap Exploris^{TM} 480
Electrospray	Nanospray Flex^{TM} Ion Source
Operation mode	data-independent
**Full Scan Properties**	
MS scan resolution	120,000
AGC target	3e6 (300%)
Maximum ion injection time for the MS scan	60 ms
Scan range	350 to 1200 m/z
Microscans	1
Polarity	positive
RF Lens	50%
Spectra data type	profile
**DIA Properties (MS2)**	
Resolution	30,000
Maximum ion injection time for the MS/MS scans	auto
Normalised AGC target	3e6 (3000%)
Spectra data type	profile
Microscans	1
Isolation window	66
Isolation window width	13 m/z
Window overlay	2 m/z
Fixed first mass	200
HCD collision energy	30%, normalised

Supplementary references

Bell, R.T., Sahakyan, H., Makarova, K.S., Wolf, Y.I., Koonin, E.V., 2024. CoCoNuTs are a diverse subclass of Type IV restriction systems predicted to target RNA. eLife 13, RP94800. https://doi.org/10.7554/eLife.94800

Cooper, L.P., Roberts, G.A., White, J.H., Luyten, Y.A., Bower, E.K.M., Morgan, R.D., Roberts, R.J., Lindsay, J.A., Dryden, D.T.F., 2017. DNA target recognition domains in the Type I restriction and modification systems of *Staphylococcus aureus*. Nucleic Acids Res. 45, 3395–3406. https://doi.org/10.1093/nar/gkx067

Goerke, C., Pantucek, R., Holtfreter, S., Schulte, B., Zink, M., Grumann, D., Bröker, B.M., Doskar, J., Wolz, C., 2009. Diversity of prophages in dominant *Staphylococcus aureus* clonal lineages. J. Bacteriol. 191, 3462–3468. https://doi.org/10.1128/JB.01804-08

Kpeli, G., Buultjens, A.H., Giulieri, S., Owusu-Mireku, E., Aboagye, S.Y., Baines, S.L., Seemann, T., Bulach, D., Silva, A.G. da, Monk, I.R., Howden, B.P., Pluschke, G., Yeboah-Manu, D., Stinear, T., 2017. Genomic analysis of ST88 community-acquired methicillin resistant *Staphylococcus aureus* in Ghana. PeerJ 5, e3047. https://doi.org/10.7717/peerj.3047
