# Supplementary figures and images for: Integrated genomic and proteomic analysis of the mouse-adapted Staphylococcus aureus strain JSNZ
Source: Curr Res Microb Sci. 2025 Oct 13;9:100489. doi: 10.1016/j.crmicr.2025.100489 (PMC12554148; doi:10.1016/j.crmicr.2025.100489)

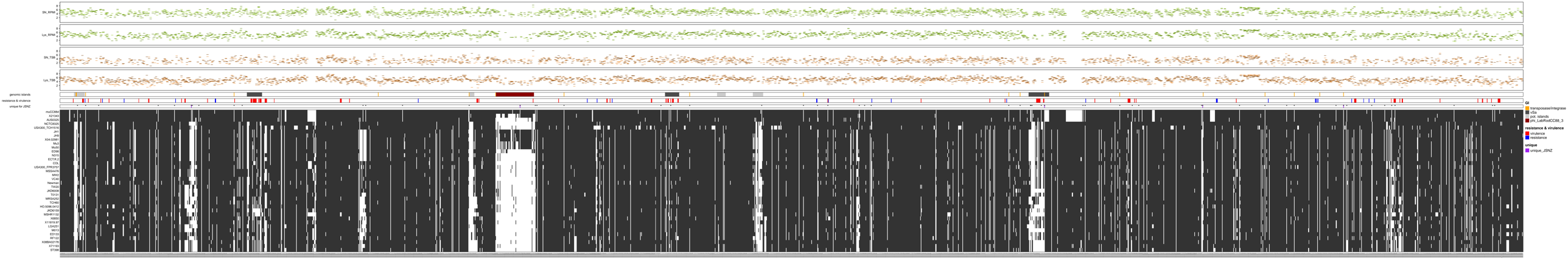

Supplement: Supplementary file 5 [file mmc5.pdf]
